# Reducing Gerontophobia and Improving Cognition: A Study on the Efficacy of Mindfulness‐Based Cognitive Therapy in Older Adults in India

**DOI:** 10.1002/alz70858_105472

**Published:** 2025-12-26

**Authors:** Pooja Rai

**Affiliations:** ^1^ Department of Clinical Psychology, School of Liberal Studies, CMR University, Bengaluru, Bengaluru, India

## Abstract

**Background:**

Gerontophobia, characterized by anxiety about aging, negatively affects mental and cognitive health in older adults. In India, where aging is often stigmatized, effective interventions are needed. Mindfulness‐Based Cognitive Therapy (MBCT) has shown promise in reducing anxiety and improving cognition. This study examines the impact of MBCT on gerontophobia and cognitive functioning in non‐demented older adults aged 50‐60 years.

**Methods:**

A quasi‐experimental pre‐test/post‐test design was employed with 35 participants divided into two groups based on Anxiety Aging Scale (AAS) scores: gerontophobia group (AAS ≥ 10, *n* = 19) and non‐gerontophobia group (AAS < 10, *n* = 16). Both groups underwent an 2‐week MBCT program. Cognitive function was assessed using the Montreal Cognitive Assessment (MoCA). Paired t‐tests and ANCOVA analyzed within and between‐group differences.

**Results:**

MBCT significantly reduced gerontophobia in the gerontophobia group (AAS: Pre *M* = 14.6, SD = 3.1; post *M* = 9.8, SD = 2.7; t(18) = ‐6.32, *p* < 0.001) and improved cognition (MoCA: Pre *M* = 24.1, SD = 3.4; post *M* = 26.5, SD = 3.1; t(18) = 6.58, *p* < 0.001). Among sub‐domains, visuospatial skills (Gerontophobia group: t(18) = 6.12, *p* < 0.001), executive functions (Gerontophobia group: t(18) = 5.28, *p* < 0.001), and attention (Gerontophobia group: t(18) = 4.76, *p* < 0.001) demonstrated statistically significant pre‐post improvement.

**Conclusion:**

MBCT effectively reduces aging‐related anxiety and enhances cognition in older adults, demonstrating its potential as a relevant intervention in India.

